# Lactoferrin Deficiency Impairs Proliferation of Satellite Cells via Downregulating the ERK1/2 Signaling Pathway

**DOI:** 10.3390/ijms23137478

**Published:** 2022-07-05

**Authors:** Xiong Wang, Fan Liu, Qin An, Wenli Wang, Zhimei Cheng, Yunping Dai, Qingyong Meng, Yali Zhang

**Affiliations:** 1 College of Food Science and Nutritional Engineering, China Agricultural University, Tsing Hua Road No. 17, Haidian District, Beijing 100083, China; b20183060522@cau.edu.cn (X.W.); anqin@cau.edu.cn (Q.A.); wenliwang82270@163.com (W.W.); green296@163.com (Z.C.); 2 State Key Laboratory for Agrobiotechnology, College of Biological Sciences, China Agricultural University, Yuanmingyuan West Road No. 2, Haidian District, Beijing 100193, China; liufanfan@cau.edu.cn (F.L.); ydai@cau.edu.cn (Y.D.); qymeng@cau.edu.cn (Q.M.)

**Keywords:** lactoferrin, skeletal muscle, satellite cells, proliferation, *ERK1/2*

## Abstract

*Lactoferrin* (*Ltf*), a naturally active glycoprotein, possesses anti-inflammatory, anti-microbial, anti-tumor, and immunomodulatory activities. Many published studies have indicated that *Ltf* modulates the proliferation of stem cells. However, the role of *Ltf* in the proliferation of satellite cells, an important cell type in muscle regeneration, has not yet been reported. Here, by using *Ltf* systemic knockout mice, we illustrate the role of *Ltf* in skeletal muscle. Results shows that *Ltf* deficiency impaired proliferation of satellite cells (SCs) and the regenerative capability of skeletal muscle. Mechanistic studies showed that *ERK1/2* phosphorylation was significantly downregulated after *Ltf* deletion in SCs. Simultaneously, the cell cycle-related proteins *cyclin D* and *CDK4* were significantly downregulated. Intervention with exogenous recombinant lactoferrin (*R-Ltf*) at a concentration of 1000 μg/mL promoted proliferation of SCs. In addition, intraperitoneal injection of *Ltf* effectively ameliorated the skeletal muscle of mice injured by 1.2% BaCl_2_ solution. Our results suggest a protective effect of *Ltf* in the repair of skeletal muscle damage. *Ltf* holds promise as a novel therapeutic agent for skeletal muscle injuries.

## 1. Introduction

Lactoferrin (*Ltf*), an 80 kDa glycoprotein containing 700 amino acids, is found in exocrine fluids such as milk, saliva, tears, and bile [1,2]. *Ltf* exerts anti-inflammatory, anti-microbial, anti-tumor, and immunomodulatory activities in mammals [3,4,5,6]. Recent studies have focused on *Ltf* as a biologically active factor that regulates cell proliferation and differentiation. Kitakaze et al. [7] found that *Ltf* led to increased cell proliferation when C2C12 is exposed to *Ltf* for only the first day. Additionally, *Ltf* promotes C2C12 differentiation and myotube hypertrophy through the low-density lipoprotein receptor mediated *ERK1/2* signaling pathway. In bone generation, *Ltf* promotes osteoblast proliferation and inhibits osteoclast formation through the *ERK1/2* and *p38* signaling pathways [8]. Additionally, *Ltf* promotes hair growth in mice through the *ERK1/2/Akt* and *Wnt* signaling pathways and stimulates the proliferation of dermal papilla cells, which play a crucial role in regulating hair follicle growth and circulation [9]. In preadipocytes, *Ltf* knockout significantly reduced adipogenesis and downregulated genes related to adipogenesis [10,11]. *Ltf* appears to act as a growth factor to enhance the proliferation of normal cells, including osteoblasts, myoblasts, and human tenocytes. However, *Ltf* plays a detrimental role in cancer cell growth. Several molecular mechanisms underlying the anticancer effects of *Ltf* have been demonstrated, including regulation of the cell cycle, promotion of apoptosis, prevention of cell migration and invasion, and immunomodulation [12,13]. In conclusion, *Ltf* appears to have a wide range of biological functions. However, the effect of lactoferrin on proliferation of SCs and its mechanism of action remain unknown.

Muscle stem cells, termed satellite cells (SCs), are located between the sarcolemma and basal lamina in a quiescent state and are essential for skeletal muscle development and regeneration [14,15]. In skeletal muscles, following intense exercise or physical injury, SCs are mobilized through a highly coordinated process. After activation, the SCs begin to proliferate and differentiate into myoblasts. Subsequently, mononuclear myoblasts differentiate and fuse with pre-existing myofibers, promoting the formation of multinucleated myotubes and myofibers [16,17,18]. The proliferation and differentiation of myogenic cells continues during birth and development. Currently, modes of chemical or physical damage to skeletal muscle, such as cardiotoxin, bupivacaine, barium chloride, freezing, and extrusion, have been used [19,20]. Based on the operability and convenience of the experiment, we used a model of 1.2% BaCl_2_-induced skeletal muscle injury to clarify the functional role of *Ltf* in mice.

The *MAPK* signaling pathway plays a crucial role in the process of cell proliferation, differentiation, stress, inflammation, and apoptosis [21,22].

*MAPK* is divided into four subfamilies: *ERK1/2*, c-Jun N-terminal kinase (*JNK*), *p38 MAPK*, and *ERK5* [23]. There is abundant evidence that the activation of ERK cascades is involved in cell cycle regulation. The eukaryotic cell cycle consists of four phases: G1, S, G2, and M, which are mediated by cyclin-dependent kinases (*CDKs*) and their regulatory cyclin subunits [24]. Cyclins are positive regulators of *CDKs*. Cyclin D transcription is dependent on *ERK* activation and nuclear retention. Cells enter the S phase, and continuous *ERK* activation is essential for maintaining the level of *cyclin D1* in the G1 phase [25]. Studies have reported that *ERK1/2* is required for myoblast proliferation and is dispensable for muscle cell fusion [26]. Zhong et al. [27] reported that the *MEK-ERK1/2* pathway is involved in muscle proliferation.

In this study, by using *Ltf* systemic knockout mice, we illustrate the role of *Ltf* in skeletal muscle. We found that *Ltf*-knockout (*Ltf*-KO) mice had poorer skeletal muscle repair capacity in response to 1.2% BaCl_2_ injury compared to normal wild-type (WT) mice. The weakened repair ability of skeletal muscle in mice is mainly due to the reduction in proliferation of SCs caused by *Ltf* deletion via the *ERK1/2* signaling pathway.

## 2. Results

### 2.1. Ltf Deficiency Impairs Regenerative Capability of Skeletal Muscle

**Figure 1:**
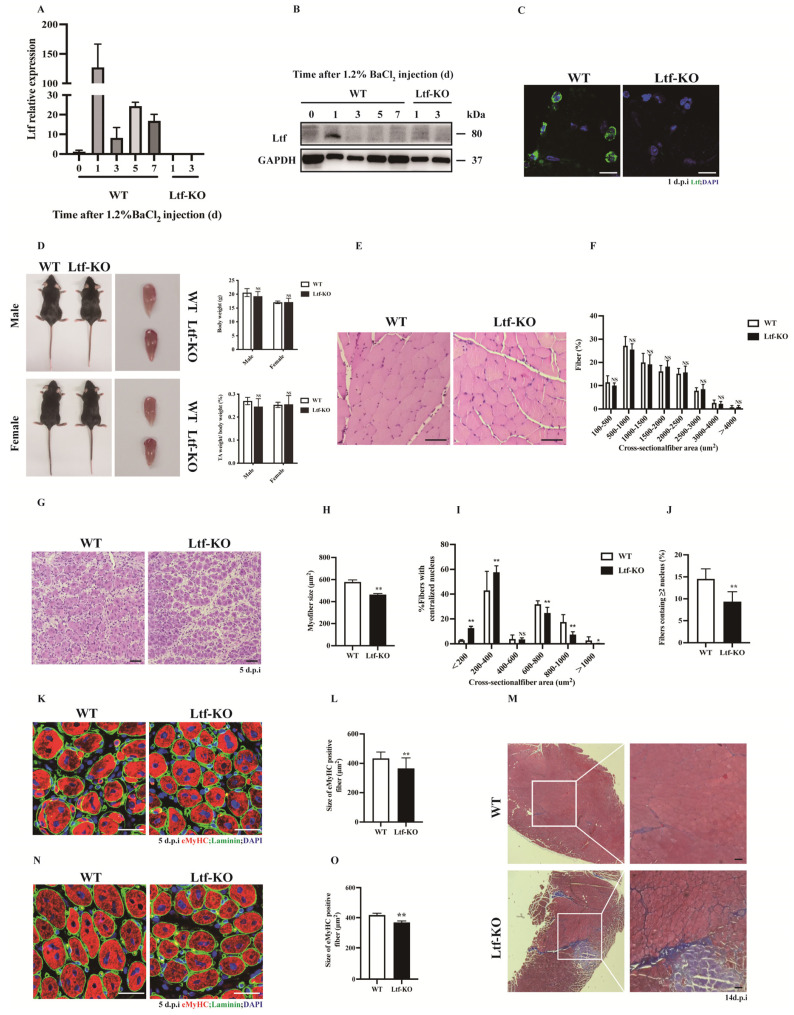
***Ltf* deficiency impairs regenerative capability of skeletal muscle.** (**A**) Real-time RT-PCR of *Ltf* expression levels in WT mice of TA muscle from days 0 to day 7 after BaCl_2_ injury and detected the TA tissue of *Ltf*-KO mice injured for 1 day and 3 days. (**B**) Immunoblots illustrate the protein levels of *Ltf* and an unrelated *GAPDH* in TA muscles from days 0 to day 7 after BaCl_2_ injury and detected the TA tissue of *Ltf*-KO mice injured for 1 day and 3 days. (**C**) Immunofluorescence results represent *Ltf* expression in WT and *Ltf*-KO mice of TA tissue on day 1 of injury. Scale bar: 10 μm. (**D**) Absolute body weight of adult WT and *Ltf*-KO mice, relative uninjured TA muscle wet weight of adult WT and *Ltf*-KO mice. (**E**) Pathological analysis plots represent hematoxylin and eosin (H&E) –stained TA cross-sections (CSA) of WT and *Ltf*-KO mice in the uninjured state. Scale bar: 60 μm. (**F**) Myofiber size (percentage) distributions of WT and *Ltf*-KO TA muscle were measured by using ImageJ software. (**G**) Representative photomicrographs of H&E-stained sections showing a delayed regeneration of injured TA muscle in *Ltf*-KO mice compared with that in WT at day 5 after BaCl_2_ injection. Scale bar: 60 μm. (**H**) Statistical analysis of mean area of TA muscle CSA in adult *Ltf*-KO and WT mice 5 days after injury. (**I**) Regenerated myofiber size (percentage) distributions of WT and Ltf-KO TA muscle 5 days after BaCl_2_ injury were measured by using ImageJ software. Only myofibers with centrally located nuclei were counted. (**J**) Quantification of the ratio of regenerating myofibers containing two or more centralized nuclei per field at day 5 post-injury. (**K**) Representative overlaid photomicrographs of TA muscle sections of WT and *Ltf*-KO mice 5 days post-injury after immunostaining for eMyHC (red) and laminin (green). Nuclei were labeled by DAPI. Scale bar: 30 μm. (**L**) Average CSA of eMyHC-positive fibers in TA muscle 5 days post-injury. (**M**) Representative photomicrographs of Masson-stained sections show increased collagen fibers in damaged TA muscles in *Ltf*-KO mice compared to WT at day 14 after BaCl_2_ injection. Scale bar: 60 μm. (**N**) At 60 days after the first injury, a second injection of BaCl_2_ solution was delivered to the muscle of WT and *Ltf*-KO mice, and the muscle was analyzed at day 5 after the second injury. Scale bar: 30 μm. (**O**) Average CSA of eMyHC-positive fibers in TA muscle at 5 days after the second injury. *n* = 3 in each group. * *p*< 0.05; ** *p* < 0.01; NS, not significant.

To determine the contribution of *Ltf* to skeletal muscle, we examined the expression profile of *Ltf* at different time points after BaCl_2_ injured tibialis anterior (TA) muscle in WT mice. The results showed that mRNA and protein levels of *Ltf* were dramatically upregulated at 1-day-post-injury (dpi) in BaCl_2_-injured TA muscle of WT mice (Figure 1A,B). We did not detect *Ltf* levels in the TA of *Ltf*-KO mice at 1 and 3 dpi (Figure 1A,B). Using immunofluorescence, we confirmed that *Ltf* was expressed at 1 dpi in the TA of WT mice (Figure 1C). Similarly, the expression of *Ltf* was not detected at 1 dpi in *Ltf*-KO mice.

Since *Ltf* was strongly upregulated after 1 dpi in BaCl_2_-injured TA muscle, this aroused great interest in exploring the role of *Ltf* in skeletal muscle. We found that *Ltf* deficiency did not affect the body weight or TA/body weight ratios in normal adult mice (Figure 1D). Additionally, the cross-sectional area (CSA) size and size distribution of TA muscles in normal adult mouse were not significantly different between WT and *Ltf*-KO mice (Figure 1E,F). Interestingly, loss of *Ltf* resulted in a significant delay in skeletal muscle repair after 5 days BaCl_2_ injury (Figure 1G). As shown in Figure 1H, the nuclear-centered muscle fiber area was significantly reduced in *Ltf*-KO mice compared to WT mice, and the number of fibers with a cross-sectional area of less than 400 μm^2^ increased significantly (Figure 1I). Moreover, compared with WT mice, the number of newly formed myofibers with two or more centralized nuclei in *Ltf*-KO mice was drastically reduced (Figure 1J). Calculation of myofiber size at 7 dpi also clearly demonstrated impaired muscle regeneration in *Ltf*-KO mice (Supplementary Figure S1A). The embryonic/developmental isoform of MyHC^+^ (eMyHC) fiber size of the TA muscle after 5 days of injury was markedly reduced in *Ltf*-KO mice compared to WT mice (Figure 1K,L). Furthermore, Masson staining showed that compared to WT mice, *Ltf*-KO mice produced more collagen after 14 days of TA muscle injury repair (Figure 1M). However, the average fiber area did not significantly different between *Ltf*-KO and WT mice after injury repair for 60 days (Supplementary Figure S1B). To further investigate the function of *Ltf* in skeletal muscle injury repair, we performed secondary injury to the TA muscle after the first round of TA injury repair for 60 days. After the secondary injury, muscle regeneration ability and the average area of new muscle fibers of *Ltf*-KO mice were significantly reduced (Figure 1N,O).

### 2.2. Deletion of Ltf Reduces Proliferative Capability of SCs

**Figure 2:**
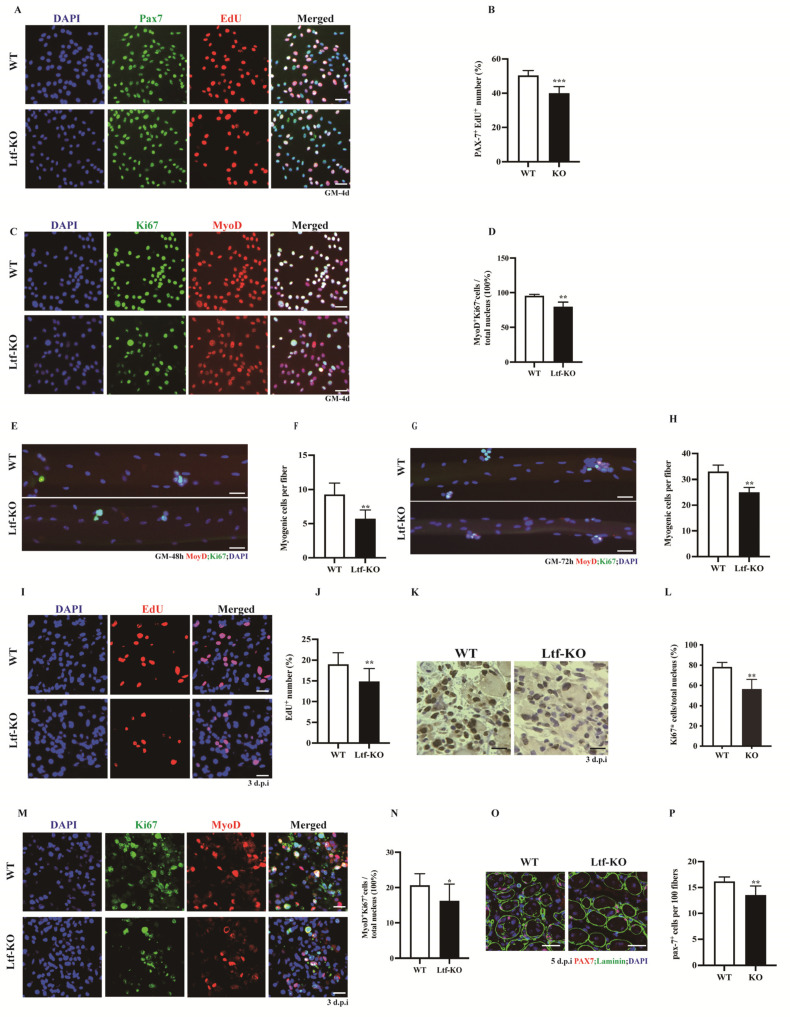
**Deletion of *Ltf* reduces proliferative capability of SCs.** (**A**) Representative individual and merged photomicrographs of SCs sorted from WT and *Ltf*-KO mice; cultured for 4 days; and labeled with EdU, Pax7, and DAPI. Scale bars: 30 μm. (**B**) Quantitative analysis of the frequency of Pax7^+^EdU^+^ double-positive cells sorted from WT and *Ltf*-KO mice. (**C**) Representative individual and merged photomicrographs of SCs sorted from WT and *Ltf*-KO mice; cultured for 4 days; and co-stained with MyoD, Ki67, and DAPI. Scale bars: 30 μm. (**D**) Quantification of the number of MyoD^+^Ki67^+^ double-positive cells sorted from WT and *Ltf*-KO mice. (**E**) Representative merged photomicrographs of 48 h cultured myofibers from WT and *Ltf*-KO mice co-stained with MyoD, Ki67, and DAPI. Scale bars: 30 μm. (**F**) Quantification of myogenic cell number per myofiber. (**G**) Representative merged photomicrographs of 72 h cultured myofibers from WT and *Ltf*-KO mice co-stained with MyoD, Ki67, and DAPI. Scale bars: 30 μm. (**H**) Quantification of myogenic cells number per myofiber. (**I**) Representative individual and merged images of day 3 post-injury TA muscles of WT and *Ltf*-KO mice, cells were labeled with EdU and DAPI. Scale bar: 30 μm. (**J**) Quantification of the number of EdU^+^ cells in day 3 post-injury TA muscles of WT and *Ltf*-KO mice. (**K**) Immunohistochemistry analysis of Ki67^+^ cells in regenerating muscle from WT and *Ltf*-KO mice at day 3 after injury. Scale bars: 30 μm. (**L**) Quantification of the number and percentage of Ki67+ cells in regenerating TA muscle of WT and *Ltf*-KO mice. (**M**) Representative individual and merged images of day 3 post-injury TA muscles of WT and *Ltf*-KO mice, cells were labeled with MyoD, Ki67and DAPI. Scale bar: 30 μm. (**N**) Quantification of the percentage of MyoD+Ki67+ double-positive cells among the total nuclei in injured TA muscle of WT and *Ltf*-KO mice. (**O**) Representative merged images of day 5 post-injury TA muscles of WT and *Ltf*-KO mice, cells were labeled with PAX7, Laminin and DAPI. Scale bar: 30 μm. (**P**) Quantification of the percentage of PAX7-positive cells among the total nuclei in injured TA muscle of WT and *Ltf*-KO mice. *n* = 3 in each group. * *p*< 0.05; ** *p* < 0.01; *** *p* < 0.001.

SCs play an important role in skeletal muscle growth and regeneration. We obtained quiescent SCs using flow cytometry and cultured them in vitro for 4 days. Cell proliferation was then assessed using EdU tracking, which showed that the number of Pax7^+^EdU^+^ SCs in *Ltf*-KO mice was significantly reduced by approximately 10% compared to that in WT controls (Figure 2A,B). Similarly, *Ltf* deletion significantly decreased the number of MyoD^+^Ki67^+^ cells relative to the WT controls (Figure 2C,D). Simultaneously, we isolated single muscle fibers from WT and *Ltf*-KO mice and cultured them for 48 and 72 h in vitro. The results showed that *Ltf* knockout led to a significant decrease in the number of MyoD^+^Ki67^+^ cells on single muscle fibers cultured for 48 (Figure 2E,F) and 72 h (Figure 2G,H). To explore whether *Ltf* affects the proliferation of SCs during skeletal muscle injury, we conducted a comprehensive analysis of the TA muscle after injury repair for 3 days. Cell tracking was performed using intraperitoneal injection of 50 mg/kg EdU at 2 h before the mice were euthanized. Compared with that in the WT mice, the number of EdU^+^ cells in the TA cross-section of *Ltf*-KO mice was significantly reduced by approximately 4% (Figure 2I,J). Consistent with these results, immunohistochemistry results showed that the number of Ki67^+^ cells in TA cross-sections of *Ltf*-KO mice was significantly reduced compared with that in WT mice (Figure 2K,L). In addition, we used MyoD to label myogenic cells and found that the loss of *Ltf* resulted in a significant reduction in the number of MyoD^+^Ki67^+^ cells (Figure 2M,N). Furthermore, relative to WT mice, the proportion of PAX7^+^ cells in the 5 dpi TA muscle of *Ltf*-KO mice was significantly reduced (Figure 2O,P).

### 2.3. Decreased Proliferation of SCs Caused by Ltf Deficiency Is Regulated by the ERK Signaling Pathway

**Figure 3:**
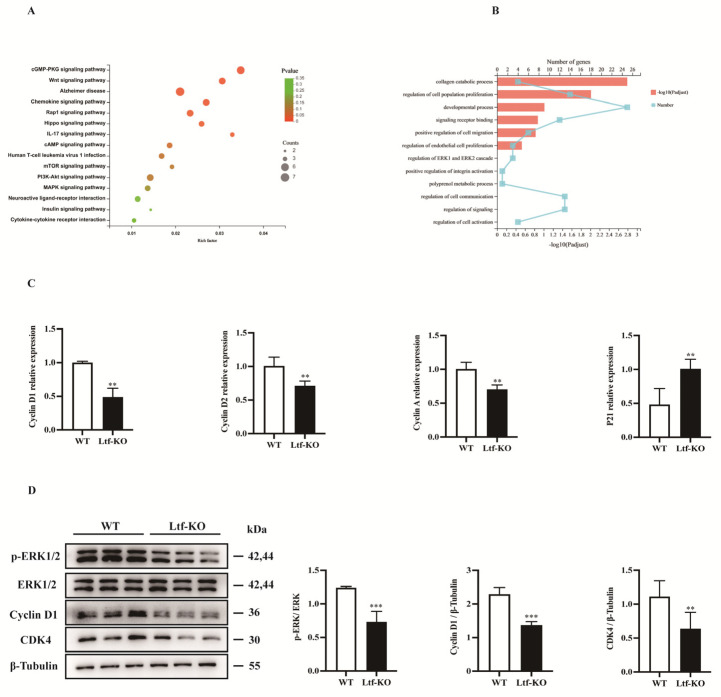
**Decreased proliferation of SCs caused by *Ltf* deficiency is regulated by the ERK1/2 signaling pathway.** (**A**) KEGG pathway enrichment analysis was performed on SCs from WT and *Ltf*-KO mice grown for 4 days in vitro. (**B**) GO enrichment analysis was performed on SCs from WT and *Ltf*-KO mice grown for 4 days in vitro. (**C**) Fluorescence quantitative analysis of the expression of cell cycle-related genes in SCs from WT and *Ltf*-KO mice grown for 4 days in vitro, using actin as an internal reference. (**D**) Left, the immunoblots presented here illustrate the protein levels of p-ERK, ERK, cyclin D, CDK4 and β-tubulin in SCs from WT and *Ltf*-KO mice grown for 4 days in vitro; right, quantification analysis of p-ERK, cyclin D1 and CDK4 proteins levels from Western blot bands. β-tubulin was used as an internal reference. *n* = 3 in each group. ** *p* < 0.01; *** *p* < 0.001.

To investigate the molecular mechanism by which *Ltf* regulates the proliferative capacity of SCs, we performed RNA sequencing analysis of SCs grown for 4 days in vitro. Using Kyoto Encyclopedia of Genes and Genomes (KEGG) and Gene Ontology (GO) enrichment analysis, we found that differential genes were enriched in the *MAPK*-*ERK1/2* signaling pathway (Figure 3A,B). We performed RT-PCR of SCs cultured for 4 days in vitro and found that cell cycle-related genes, such as those for *cyclin D1*, *cyclin D2*, and *cyclin A*, were significantly downregulated after *Ltf* deletion and *Ltf* deletion led to upregulation of *p21* (Figure 3C). In addition, Western blotting results showed that *p-ERK*, *cyclin D*, and CDK4 protein levels were significantly downregulated after *Ltf* deletion (Figure 3D).

### 2.4. R-Ltf Promotes Damage Repair of Skeletal Muscle

**Figure 4:**
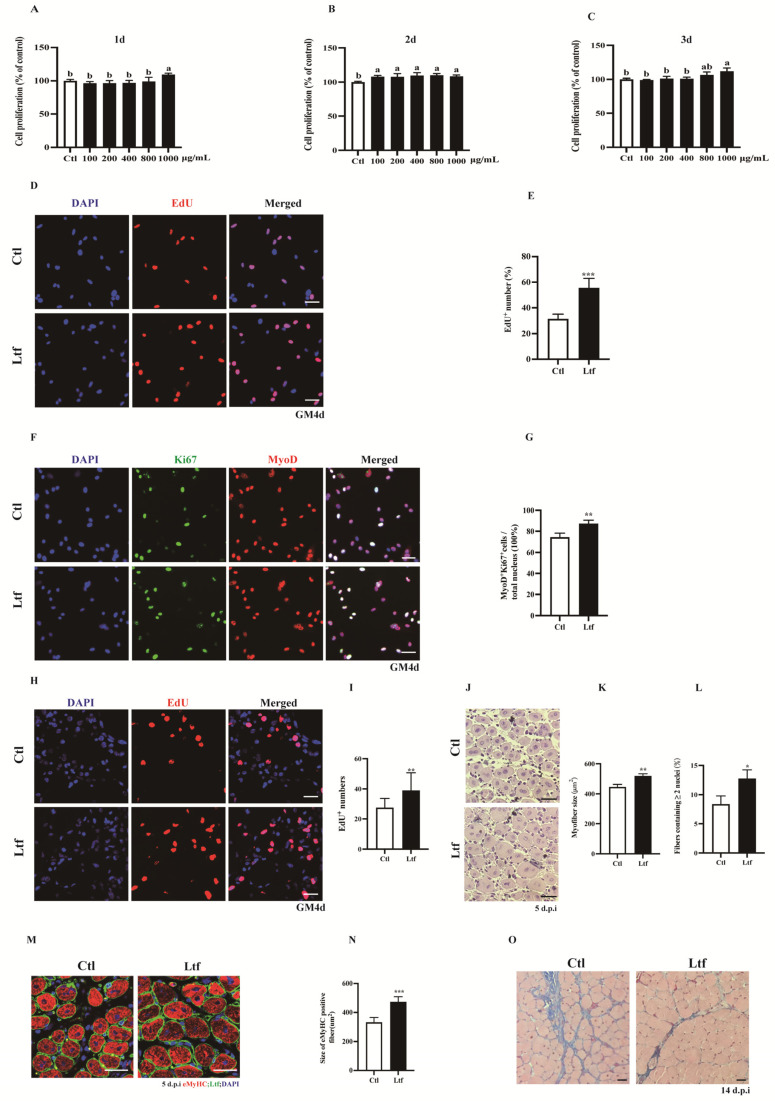
**R-*Ltf* promotes proliferation of SCs in vivo and in vitro.** (**A**) Satellite cells (SCs) isolated from wild-type (WT) mice after 1 day of adherent growth, treated with various concentrations of R-*Ltf* for 1 day, and then added CCK-8 to determine the OD value. (**B**) SCs isolated from WT mice after 1 day of adherent growth, treated with various concentrations of R-*Ltf* for 2 days, and then added CCK-8 to determine the OD value. (**C**) SCs isolated from WT mice after 1 day of adherent growth, treated with various concentrations of R-*Ltf* for 3 days, and then added CCK-8 to determine the OD value. (**D**) SCs isolated from WT mice after 1 day of adherent growth, treated with 1000 μg/mL R-*Ltf* for other 3 days. Cells were labeled with EdU and DAPI. Scale bar: 20 μm. (**E**) Quantitative analysis of the frequency of EdU+ positive cells. (**F**) SCs isolated from WT mice after 1 day of adherent growth and treated with 1000 μg/mL R-*Ltf* for other 3 days. Cells were labeled with MyoD, Ki67 and DAPI. Scale bar: 20 μm. (**G**) Quantification of the percentage of MyoD^+^Ki67^+^ double-positive cells. (**H**) Representative individual and merged photomicrographs of day 3 post-injury TA muscles of WT; following consecutive intraperitoneal injection of R-*Ltf* for 3 days, the cells were labeled with EdU and DAPI. Scale bars: 20 μm. (**I**) Quantitative analysis of the frequency of EdU+ positive cells. (**J**) Representative photomicrographs of hematoxylin and eosin (H&E)-stained sections of injured TA muscle of WT at day 5 after BaCl_2_ injection, with consecutive intraperitoneal injection of R-*Ltf* for 5 days. Scale bar: 30 μm. (**K**) Quantification of the ratio of regenerating myofibers containing two or more centralized nuclei per field at day 5 post-injury. (**L**) Statistical analysis of mean area of TA muscle cross-sections (CSA) in adult WT mice 5 days after injury. (**M**) Representative overlaid photomicrographs of TA muscle sections of WT mice 5 days post-injury, followed by consecutive intraperitoneal injection of R-*Ltf* for 5 days, after immunostaining for eMyHC (red) and laminin (green). Nuclei were labeled by DAPI. Scale bar: 30 μm. (**N**) Average CSA of eMyHC-positive fibers in TA muscle 5 days post-injury. (**O**) Representative photomicrographs of Masson-stained sections show collagen fibers in damaged TA muscles in WT mice at day 14 after BaCl_2_ injection, intraperitoneal injection of R-*Ltf* for 14 days. Scale bar: 30 μm. *n* = 6 in each group. * *p*< 0.05; ** *p* < 0.01; *** *p* < 0.001. Values without a common letter are significantly different at *p* < 0.05.

To investigate the effect of exogenous addition of *R-Ltf* on SCs proliferation, we used a concentration gradient of R-*Ltf* to treat in vitro cultured SCs. We found that at a concentration of 1000 μg/mL, *R-Ltf* better promoted the proliferation of SCs at 1, 2, and even 3 days (Figure 4A–C). In addition, compared to the control group, the number of EdU^+^ cells increased by approximately 25% after 1000 μg/mL *R-Ltf* intervention for 3 days (Figure 4D,E). Similarly, 1000 μg/mL *R-Ltf* treatment of SCs increased the number of MyOD^+^Ki67^+^ cells relative to that in the control group (Figure 4F,G). In vivo, we administered 100 mg/kg dose of *R-Ltf* to TA muscle-injured mice via intraperitoneal injection for 3 consecutive days. The results showed that *R-Ltf* effectively increased the number of EdU^+^ cells in the TA muscle repaired for 3 days (Figure 4H,I). In addition, we analyzed the cross-sectional area of the 5-day TA muscle after injury repair. Compared with the control group, the CSA of the TA muscle fibers in the *R-Ltf*-treated group was larger (Figure 4J,K). Similarly, the proportion of new myotubes with ≥ 2 nuclei in the TA muscle of the R-*Ltf*-treated group was significantly higher than that in the control group (Figure 4L). In addition, the area of eMyHC^+^ positive new myotubes in the R-*Ltf*-treated group was significantly larger than that in the control group (Figure 4M,N). *R-Ltf* treatment effectively reduced collagen fiber production in the TA muscle for 14 days after repair relative to that in the control group (Figure 4O).

## 3. Discussion

The anti-inflammatory, anti-tumor, and antibacterial properties of exogenous *Ltf* have been widely reported [28,29]. Recently, an increasing number of studies have focused on the functional role of *Ltf* in cell proliferation and biological tissue regeneration. Studies have shown that *Ltf* promotes osteoblast [30], hair [9], and intestinal cell [31] proliferation. In addition, *Ltf* significantly increased the proportion of wound re-epithelialization during skin burn healing [32]. Some studies have reported that *Ltf* deficiency in mice promotes metastasis of melanoma cells to the lung by downregulating the TLR9 signaling pathway to recruit bone marrow-derived suppressor cells [33]. Studies have shown that *Ltf* is necessary for the early stage of mouse B-cell development, and that the absence of *Ltf* provides a less favorable microenvironment for early B-cell development [34]. These studies suggest that *Ltf* acts as a growth factor to regulate the growth of various cells directly or indirectly, thereby participating in the regeneration of biological tissues. However, the function of *Ltf* in skeletal muscles remains unknown. We found that *Ltf* levels were drastically upregulated at 1 day after BaCl_2_ injury to the TA muscle. The immunofluorescence results also proved that *Ltf* was expressed in the TA muscle 1 day after BaCl_2_ injury to the TA muscle. This aroused our interest in exploring the role *Ltf* plays in skeletal muscles. Therefore, we constructed *Ltf* systemic knockout mice to investigate the role of *Ltf* in skeletal muscle regeneration.

Muscle injury repair includes three sequential and programmed processes: (1) the inflammatory response; (2) SC activation, proliferation, differentiation, and fusion; and (3) muscle fiber maturation and remodeling of new muscle fibers [35,36,37]. Pathological analysis showed that *Ltf* knockout delayed the repair of damaged skeletal muscle, manifested as a smaller area of new muscle fibers and more collagen fiber formation compared to WT control mice. Skeletal muscle injury repair and regeneration involve an important group of cells, known as SCs, located between the basement membrane and sarcolemma [38,39]. Numerous cell types proliferate at 3 days after injury repair and involved in regeneration [40]. We found that compared with WT mice, the proportion of proliferating cells in the 3-day-repaired TA in *Ltf*-KO mice was significantly reduced. To confirm whether the myogenic cell population showed decreased proliferation, we used MyoD to label myogenic cells. We found that the proportion of MyoD^+^ Ki67^+^ double-positive cells in the 3-day-repaired TA tissue of *Ltf*-KO mice was significantly reduced relative to that in WT mice (Figure 2M,N). In addition, our in vitro experiments with SCs (Figure 2C,D) and isolated single muscle fibers (Figure 2E–H) also showed that the proportion of MyoD^+^ Ki67^+^ double-positive cells was significantly reduced after *Ltf* depletion compared to that in the WT control. Thus, the lack of *Ltf* impairs the proliferation of SCs and delays the injury repair in skeletal muscle of *Ltf*-KO mice.

We next examined how the loss of *Ltf* mediates the reduction in the proliferative capacity of SCs. The involvement of *Ltf* in regulating of cell proliferation has been widely reported. *Ltf* acts as a positive regulator of normal cell proliferation while exerting an inhibitory effect on cancer cells [13,41]. It is well-known that the *ERK* signaling pathway is involved in the regulation of cell proliferation. Evidence suggests that *ERK* pathway activation promotes cell cycle entry from G1 to S phase by inducing of *cyclin D* and assembly of *cyclin D*–*CDK4* complexes [42,43,44]. Sustained *ERK* activation is critical for maintaining *cyclin D* levels in the G1 phase for cells to enter the S phase [45]. Many studies confirmed that the *ERK1/2* signaling pathway plays an important role in regulating the proliferation of SCs and myoblasts [26,27,46]. Additionally, *Ltf* partially affects C2C12 cell proliferation and differentiation through the *Lrp1* receptor-mediated *ERK1/2* signaling pathway [7]. Studies have reported that *Ltf* plays an important role in bone [47], hair [9] and skin regeneration [40], which is mainly achieved by mediating the *ERK* signaling pathway. Certainly, *Ltf* mediates the regulation of cell proliferation through other signaling pathways. Studies have reported that *Ltf* mediates osteoblast proliferation through the LRP1-independent *p38* signaling pathway [8,48]. In addition, Liu et al. [49] showed that bovine-*Ltf* promotes human intestinal epithelial cell proliferation by activating the *PI3K/Akt* signaling pathway. We performed KEGG and GO enrichment analyses on RNA-seq and found that differentially expressed genes were enriched in the *ERK* pathway. Analysis of SCs obtained using flow sorting for 4 days, and the results showed that *Ltf* knockout significantly downregulated the protein levels of *p-ERK1/2*, *cyclin D* and *CDK4* (Figure 3D). Moreover, we also detected the phosphorylation levels of *AKT, JNK* and *p38* showed no significant changes after *Ltf deletion* (Supplementary Figure S2). Thus, the reduction in the proliferation capacity of SCs caused by *Ltf* deficiency is regulated by the *ERK1/2* signaling pathway, at least in part.

As *Ltf* plays a key role in maintaining the proliferation of SCs, we explored whether exogenous *Ltf* intervention could act as a functional factor to regulate the proliferation of SCs and help repair skeletal muscle damage. To verify the effect of *Ltf* on the proliferation ability of SCs, we treated the SCs with various concentrations of exogenous *R-Ltf*. It has been reported that exogenous *Ltf* promotes the proliferation of C2C12 cells and myogenic differentiation, thereby promoting myotube hypertrophy [7]. Zhang et al. [50] showed that *Ltf* promoted human tenocyte proliferation and collagen synthesis at a concentration of 100 µg/mL. Similar to previously published literature, our in vitro results showed that *R-Ltf* at 1000 μg/mL promoted SC proliferation compared with control (Figure 4A-C). In addition, 100 mg/kg *R-Ltf* effectively alleviated BaCl_2_ injury in the TA muscles of WT mice. Compared to the control group, intraperitoneal injection of *R-Ltf* for 3 days significantly increased the number of EdU^+^ cells (Figure 4H,I). Moreover, histopathological image analysis of TA also showed that intraperitoneal injection of *R-Ltf* helped repair damaged skeletal muscles. The area of new muscle fibers (Figure 4J,M) in the intraperitoneal injection of *R-Ltf* treatment group was larger, and the formation of collagen fibers (Figure 4O) was lower than that in the control group. Whether *Ltf* is an effector molecule to regulate the proliferation of SCs requires further analysis.

In conclusion, *Ltf* may act as a functional factor that maintains the proliferation of SCs. *Ltf* depletion impaired the proliferation of SCs by downregulating *ERK1/2* signaling. In addition, intraperitoneal injection of *R-Ltf* effectively helped in the repair and renewal of skeletal muscle after injury, providing evidence for the development of a therapeutic approach in medical and biological applications.

## 4. Materials and Methods

### 4.1. Materials

Recombinant lactoferrin (purity ≥ 99%) was donated by Professor Dai Yunping’s laboratory at the Life Science Center of the China Agricultural University. Collagenase I (Cat. No:C6885), collagenase II (Cat. No:C0130), and b chloride (BaCl_2_) powder were purchased from Sigma-Aldrich (St. Louis, MO, USA). Dispase II (Cat. No:04942078001) was purchased from Roche (Basel, Switzerland). Dulbecco’s Modified Eagle Medium (DMEM) (Cat. No:11965118), Ham’s F10 medium (Cat. No:11550043), fetal bovine serum (FBS) (Cat. No:10091-148), horse serum (Cat. No:26050088), trypsin-EDTA (0.25%) (Cat. No:25200072), penicillin-streptomycin (10,000 U/mL) (Cat. No:15140163), and Dulbecco’s phosphate-buffered saline (DPBS) (Cat. No:14190144) were purchased from Gibco (Grand Island, NY, USA). Chicken embryo extract (Cat. No:100-163P) was purchased from GeminiBio (West Sacramento, CA, USA). Recombinant human fibroblast growth factor (FGF) (Cat. No:13256-029) was purchased from Invitrogen (Carlsbad, CA, USA). CCK-8 Kit was purchased from LABLEAD (Beijing, China).

### 4.2. Generation of Ltf Knockout Mice

The *Ltf* gene is located on the positive strand of chromosome 9, with a total length of approximately 23.5 kb. To evaluate the functional role of *Ltf* in skeletal muscle, exon 2–8 fragments of the gene were knocked out in C57/BL6N mouse zygotes using CRISPR-Cas9 technology to generate a systemic *Ltf* knockout model. Single-guide RNA sequences were designed in Introns 2 and 8, resulting in a genome deletion of approximately 4 kb, to knock out the *Ltf* gene. The specific gene-targeting strategies and single-guide RNA sequences are shown in Supplementary Figure S3A and Supplementary Table S1. The mouse tail genome was subjected to PCR using the primers listed in Supplementary Table S2, and the agarose gel results of the products are shown in Supplementary Figure S3B.

### 4.3. Tibialis Anterior Muscle Injury

All animal experiments were performed according to the Animal Care and Use Committee and guidelines for animal experiments at China Agricultural University (Aw02602202-3-1, 24 February 2020). All animals were kept in a temperature- and humidity-controlled facility (20 ± 2 °C and 50% relative humidity, with a 12 h light/dark cycle, ammonia concentration less than 20 ppm, air velocity 10–25 cm/s, the number of air change 10–15 times per hour) with free access to water and a chow diet. The mice used in the experiments were all 8–10 weeks old, unless otherwise specified.

The mouse TA muscle was injured as described previously [51]. Briefly, after the mice were anesthetized with an anesthesia machine, 75 μL of sterile 1.2% BaCl_2_ solution was injected into the center of the TA muscle at a fixed point, and the samples were collected at the various time points for analysis.

### 4.4. SCs Isolation and Culture

WT and *Ltf*-KO mice were euthanized, and the skin was disinfected with 75% alcohol. The mouse skin was cut using sterile scissors to fully expose the leg muscles. Using additional sterile scissors and forceps, the hind leg muscles were completely removed and placed in sterile ice-cold DMEM. The tissue was cut into as many pieces as possible. The minced tissue was digested with collagenase II (500 U/mL) in DMEM containing 1% penicillin–streptomycin at 37 °C on a horizontal shaker for 40 min. The digested cell tissue suspension was collected using centrifugation at 1500× *g*, 5 min, 4 °C and washed with ice-cold DPBS. Further digestion was performed with collagenase II (200 U/mL) and dispase II (2.4 U/mL) at 37 °C on a horizontal shaker for 30 min. Single cells were obtained by filtering the digested cell suspension through a 40 µm nylon cell strainer and washed twice with ice-cold sterile DPBS. Single cells were stained with CD31-PE/Cy7 (clone 390; BioLegend, San Digeo, CA, USA), CD45-FITC (clone 30-F11; BD Biosciences, Franklin Lakes, NJ, USA), Sca1-PerCp (clone D7; eBioscience, San Digeo, CA, USA), and α-integrin-7-APC (clone R2F2; AbLab, Vancouver, Canada) on ice for 1 h in the dark. SCs were sorted using a Beckman cell sorter equipped with lasers (Brea, CA, USA). Sca1^−^/CD11b^−^/CD34^+^/α7-integrin^+^ SCs were collected into sterile tubes containing 500 μL of DMEM supplemented with 20% fetal bovine serum, 1% chicken embryo extract, 10 ng/mL basic fibroblast growth factor, and 1% penicillin–streptomycin at 4 °C. For growth, sorted cells were placed on 10% Matrigel-pre-coated (BD Biosciences) cell culture plates at 37 °C in a 5% CO_2_ atmosphere.

### 4.5. Myofiber Isolation and Culture

The mice were euthanized, and the skin was disinfected with 75% alcohol. Mouse skin was cut with sterile scissors to fully expose the leg muscles, and the cervical membrane was removed. The TA and extensor digitorum longus (EDL) were completely separated by gently sliding the tendon using ophthalmic surgical forceps and cutting at the base of the tendon with a scalpel. The tendon was gently picked up with tweezers and cut at the muscle below the knee joint to avoid stretching the muscle. Finally, TA and EDL were separated using tweezers and placed in ice-cold DMEM for later use. The separated EDL was digested in 0.2% collagenase Ⅰ in serum-free DMEM at 37 °C on a horizontal shaker for 1 h. For suspension culture, single myofibers were grown on plates pre-coated with horse serum. For growth, single myofibers were cultured in DMEM supplemented with 10% horse serum, 1% chicken embryo extract, and 1% penicillin–streptomycin. Single myofibers were cultured at 37 °C, 5% CO_2_ atmosphere for 48 or 72 h.

### 4.6. Cell Proliferation Assay

In vivo cell and SC proliferation assays were evaluated using the Click-iT EdU Cell Proliferation Assay Kit (Invitrogen). To assess in vivo cell proliferation, 50 mg/kg body weight EdU was injected intraperitoneally for 2 h prior to harvesting the regenerating TA muscles from WT and *Ltf*-KO mice at 3 days post-injury. For SC proliferation assay, 2.5 × 10^4^ SCs were seeded into 10% Matrigel-pre-coated 24-well cell culture plates and incubated at 37 °C in a 5% CO_2_ atmosphere for 4 days. For the *R-Ltf* intervention experiment, 1000 µg/mL *R-Ltf* was added and incubated with the cells for 3 days after the SCs adhered for 24 h. SCs were tracked with 10 mM EdU for 2 h before harvesting. The old cell culture medium was discarded, and the cells were washed twice with DPBS and fixed in 4% paraformaldehyde (PFA) for 15 min at room temperature. An EdU test was performed according to the manufacturer’s instructions.

### 4.7. Histological and Morphometric Analysis

To assess the morphology and regeneration of the injured TA muscle, the 5-day TA-injured muscle was collected and fixed in 4% paraformaldehyde, followed by procedural dehydration and paraffin embedding. The processed paraffin tissue blocks were sectioned at a thickness of 5 μm using a Leica microtome (Wetzlar, Germany). Paraffin sections were deparaffinized programmatically and stained using the H&E staining kit. The cross-sectional area of the hematoxylin-eosin-stained TA muscle was calculated using ImageJ software (NIH, Bethesda, MD, USA).

### 4.8. qRT-PCR Analysis

Total RNA was extracted from TA muscle and SCs using TRIzol reagent (Invitrogen, Life Technologies) and purified using the UNIQ-10 Column Total RNA kit (BBI Life Science, Shanghai, China) according to the manufacturer’s standards. The RNA concentration was determined by measuring the A260/A280 and A260/A230 ratios using a BioDrop μLITE (BioDrop, Cambridge, UK). Total RNA (1 μg, 10 μL) was reverse-transcribed using an M-MuLV cDNA Synthesis Kit (BBI Life Science, Shanghai, China). qRT-PCR was performed using 2× SG Fast qPCR Master Mix (BBI Life Science, Shanghai, China). RT-PCR amplification conditions included denaturation at 95 °C for 2 s, annealing at 60 °C for 25 s, and extension for 30 s at 72 °C. Relative quantification of the target gene were calculated using the 2^−∆∆CT^ method, with reference to the control diet group, with GAPDH as a control. The sequencing primers used in this study are listed in Table 1.

### 4.9. RNA Sequencing

SCs obtained by flow sorting were seeded in 6-well cell culture plates at a density of 2.5 × 10^5^. After 4 days of growth, the cells were harvested, treated with 1 mL Trizol, snap-frozen in liquid nitrogen, and transported to Gene Denovo Biotechnology Co (Guangzhou, China). Data were extracted and normalized according to the manufacturer’s protocol. The RNA-seq raw expression files and details have been deposited in the NCBI GEO under accession number GSE205136. Log-fold changes in up/downregulated genes in *Ltf*-KO mice were selected with a significance threshold of *p* < 0.05. Gene ontology (GO) and KEGG pathway enrichment analyses of differentially expressed genes were performed using R based on the hypergeometric distribution.

### 4.10. Western Blotting

Proteins were extracted with RIPA lysis buffer (Cell Signaling Technology, Danvers, MA, USA, Cat. No:9806) supplemented with protease inhibitor (Beyotime Biotechnology, Jiangsu, China, Cat. No:ST507) and phosphatase inhibitor (Roche, Basel, Switzerland, Cat. No:4906845001). Protein samples and the loading buffer were mixed at a ratio of 4:1 and boiled for 10 min. After separation by SDS-PAGE (8–10%), the protein strip was transferred to a nitrocellulose membrane. The membranes were blocked using 5% non-fat milk powder in Tris-buffered saline and Tween (TBST) and incubated with primary antibodies at the indicated dilutions overnight at 4 °C. The following antibodies were purchased from Cell Signaling Technology (Danvers, MA, USA): anti-*p-ERK1/2* (Cell Signaling Technology, Cat. No:4370), anti-*ERK1/2* (Cell Signaling Technology, Cat. No:4695), anti-*cyclin D1* (Cell Signaling Technology, Cat. No:2978), anti-*CDK4* (Cell Signaling Technology, Cat. No:12790), and anti-*β-tubulin* (Cell Signaling Technology, Cat. No:2146). After washing three times with TBST buffer, the membrane was incubated for 1 h with horseradish peroxidase-conjugated anti-rabbit IgG (Cell Signaling Technology, Cat. No:7074) or anti-rabbit IgG (Cell Signaling Technology, Cat. No:91196) secondary antibody. Protein bands were detected using enhanced chemiluminescence reagent, and densitometry was performed using the ImageJ software. The band intensity of each target protein was normalized to that of β-tubulin.

### 4.11. Immunofluorescence and Immunohistochemistry

For immunostaining analysis, paraffin sections were deparaffinized programmatically, and antigen retrieval was performed on deparaffinized sections using an improved citrate antigen retrieval solution (Beyotime Biotechnology, Jiangsu, China, Cat. No: P0083) at high temperature and pressure (timed for 10 min after the pressure cooker was pressed). Paraffin sections, 4% paraformaldehyde-fixed single myofibers, and cultured SCs were washed with DPBS and permeabilized with 0.5% Triton X-100 for 15 min at room temperature. The samples were blocked with an immune-blocking solution at room temperature for 1 h and then incubated with primary antibodies at 4 °C overnight. The samples were washed four times with DPBS and incubated with secondary antibodies for 1 h at room temperature. DAPI (Sigma, St. Louis, MO, USA, Cat. No: D9542) staining was used to detect the nuclei. The following primary antibodies were used: rabbit anti-Ki67 (Invitrogen, Cat. No:PA5-114437), mouse anti-Pax7 (Developmental Studies Hybridoma Bank, Iowa City, IA, USA; Cat. No: AB_528428), mouse anti-MyoD (Santa Cruz Biotechnology, Dallas, TX, USA; Cat. No:sc-32758), mouse anti-embryonic myosin heavy chain BF-45/F1.652 (Developmental Studies Hybridoma Bank, Cat. No: AB_528358), and rabbit anti-laminin (Sigma, St. Louis, MO, USA, Cat. No: L9393).

### 4.12. CCK-8 Assay

SCs obtained using flow sorting were seeded in 96-well cell culture plates at a density of 2 × 10^3^. After the cells had adhered for 24 h, different concentrations of *R-Ltf* (configured with DMEM containing 0.5% BSA) were added, and the cells were incubated for 1, 2, and 3 days, 0.5% BSA as a control group (Ctl). Detection was conducted in strict accordance with the instructions of the CCK-8 kit. Finally, the absorbance was read using a microplate reader at a wavelength of 450 nm.

### 4.13. Intraperitoneal Injection of R-Ltf in Intervention of BaCl_2_ Injury to TA

Ten-week-old adult WT mice were TA injured, as described in Section 4.3. A 100 mg/kg dose of *R-Ltf* (configured with saline containing 0.5% BSA) was intraperitoneally injected, and the control group was intraperitoneally injected with the same volume saline containing 0.5% BSA (Ctl). TA muscle samples were collected for analysis after continuous injection for 3, 5, or 14 days.

### 4.14. Statistical Analysis

Results are presented as the mean ± standard deviation. Statistical significance was determined using one-way analysis of variance followed by Tukey’s multiple comparison test using SPSS version 19.0 (SPSS, Inc., Chicago, IL, USA). Statistical significance was set at *p* < 0.05.

## Figures and Tables

**Table 1:** Sequences of primer used for qRT-PCR.

Primer	Sequence (5′-3′)	Access NO.
Cyclin A-F	GCCCTGGCTTTTAATGCAGC	NM_009828.3
Cyclin A-R	AACGTTCACTGGCTTGTCTTC	
Cyclin D1-F	ATTGTGCCATCCATGCGGAA	NM_001379248
Cyclin D1-R	GAAGACCTCCTCTTCGCACT	
Cyclin D2-F	CTGCGGAAAAGCTGTGCATT	NM_009829
Cyclin D2-R	GAAGTCGTGAGGGGTGACTG	
p21-F	TAAGGACGTCCCACTTTGCC	NM_001111099.2
p21-R	GACAACGGCACACTTTGCTC	
β-Actin-F	TTTGCAGCTCCTTCGTTGCC	NM_007393.5
β-Actin-R	CCCACGATGGAGGGGAATACA	

## Data Availability

The data presented in this article or supplementary material are available.

## Supplementary Materials

The following supporting information can be downloaded at: https://www.mdpi.com/article/10.3390/ijms23137478/s1.

## References

[1] Superti F. (2020). Lactoferrin from Bovine Milk: A Protective Companion for Life. Nutrients.

[2] Gleerup H.S., Jensen C.S., Høgh P., Hasselbalch S.G., Simonsen A.H. (2021). Lactoferrin in cerebrospinal fluid and saliva is not a diagnostic biomarker for Alzheimer’s disease in a mixed memory clinic population. EBioMedicine.

[3] Telang S. (2018). Lactoferrin: A Critical Player in Neonatal Host Defense. Nutrients.

[4] García-Montoya I., Salazar-Martínez J., Arévalo-Gallegos S., Sinagawa-García S., Rascón-Cruz Q. (2012). Expression and characterization of recombinant bovine lactoferrin in *E. coli*. BioMetals.

[5] Wang B., Timilsena Y.P., Blanch E., Adhikari B. (2019). Lactoferrin: Structure, function, denaturation and digestion. Crit. Rev. Food Sci. Nutr..

[6] Antoshin A.A., Shpichka A.I., Huang G., Chen K., Lu P., Svistunov A.A., Lychagin A.V., Lipina M.M., Sinelnikov M.Y., Reshetov I.V. (2021). Lactoferrin as a regenerative agent: The old-new panacea?. Pharmacol. Res..

[7] Kitakaze T., Oshimo M., Kobayashi Y., Ryu M., Suzuki Y.A., Inui H., Harada N., Yamaji R. (2018). Lactoferrin promotes murine C2C12 myoblast proliferation and differentiation and myotube hypertrophy. Mol. Med. Rep..

[8] Zhang W., Guo H., Jing H., Li Y., Wang X., Zhang H., Jiang L., Ren F. (2013). Lactoferrin Stimulates Osteoblast Differentiation Through PKA and p38 Pathways Independent of Lactoferrin’s Receptor LRP1. J. Bone Miner. Res..

[9] Huang H.-C., Lin H., Huang M.-C. (2019). Lactoferrin promotes hair growth in mice and increases dermal papilla cell proliferation through Erk/Akt and Wnt signaling pathways. Arch. Dermatol. Res..

[10] Moreno-Navarrete J.M., Ortega F.J., Moreno M., Serrano M., Ricart W., Fernández-Real J.M. (2014). Lactoferrin gene knockdown leads to similar effects to iron chelation in human adipocytes. J. Cell. Mol. Med..

[11] Moreno-Navarrete J.M., Ortega F.J., Ricart W., Fernández-Real J.M. (2009). Lactoferrin increases 172ThrAMPK phosphorylation and insulin-induced p473SerAKT while impairing adipocyte differentiation. Int. J. Obes..

[12] Cutone A., Rosa L., Ianiro G., Lepanto M.S., Di Patti M.C.B., Valenti P., Musci G. (2020). Lactoferrin’s Anti-Cancer Properties: Safety, Selectivity, and Wide Range of Action. Biomolecules.

[13] Zhang Y., Lima C.F., Rodrigues L.R. (2014). Anticancer effects of lactoferrin: Underlying mechanisms and future trends in cancer therapy. Nutr. Rev..

[14] Kowalski K., Kolodziejczyk A., Sikorska M., Płaczkiewicz J., Cichosz P., Kowalewska M., Stremińska W., Jańczyk-Ilach K., Koblowska M., Fogtman A. (2016). Stem cells migration during skeletal muscle regeneration—The role of Sdf-1/Cxcr4 and Sdf-1/Cxcr7 axis. Cell Adhes. Migr..

[15] Musarò A., Carosio S. (2017). Isolation and Culture of Satellite Cells from Mouse Skeletal Muscle. Mol. Biol..

[16] Said R.S., Mustafa A.G., Asfour H.A., Shaqour E.I. (2016). Myogenic Satellite Cells: Biological Milieu and Possible Clinical Applications. Pak. J. Biol. Sci..

[17] Laumonier T., Menetrey J. (2016). Muscle injuries and strategies for improving their repair. J. Exp. Orthop..

[18] Smith H.K., Maxwell L., Rodgers C.D., McKee N.H., Plyley M.J. (2001). Exercise-enhanced satellite cell proliferation and new myonuclear accretion in rat skeletal muscle. J. Appl. Physiol..

[19] Tierney M.T., Sacco A. (2016). Inducing and Evaluating Skeletal Muscle Injury by Notexin and Barium Chloride. Methods Mol. Biol..

[20] Su W.-H., Wang C.-J., Fu H.-C., Sheng C.-M., Tsai C.-C., Cheng J.-H., Chuang P.-C. (2019). Human Umbilical Cord Mesenchymal Stem Cells Extricate Bupivacaine-Impaired Skeletal Muscle Function via Mitigating Neutrophil-Mediated Acute Inflammation and Protecting against Fibrosis. Int. J. Mol. Sci..

[21] Sun Y., Liu W.-Z., Liu T., Feng X., Yang N., Zhou H.-F. (2015). Signaling pathway of MAPK/ERK in cell proliferation, differentiation, migration, senescence and apoptosis. J. Recept. Signal Transduct..

[22] Feng Y., Hua X., Niu R., Du Y., Shi C., Zhou R., Chen F.-H. (2019). ROS play an important role in ATPR inducing differentiation and inhibiting proliferation of leukemia cells by regulating the PTEN/PI3K/AKT signaling pathway. Biol. Res..

[23] Guo Y.J., Pan W.W., Liu S.B., Shen Z.F., Xu Y., Hu L.L. (2020). ERK/MAPK signalling pathway and tumorigenesis. Exp. Ther. Med..

[24] Pokrass M.J., Ryan K.A., Xin T., Pielstick B., Timp W., Greco V., Regot S. (2020). Cell-Cycle-Dependent ERK Signaling Dynamics Direct Fate Specification in the Mammalian Preimplantation Embryo. Dev. Cell.

[25] Mercer K., Giblett S., Oakden A., Brown J., Marais R., Pritchard C. (2005). A-Raf and Raf-1 work together to influence transient ERK phosphorylation and Gl/S cell cycle progression. Oncogene.

[26] Jones N.C., Fedorov Y.V., Rosenthal R.S., Olwin B.B. (2001). ERK1/2 is required for myoblast proliferation but is dispensable for muscle gene expression and cell fusion. J. Cell. Physiol..

[27] Zhong R., Miao R., Meng J., Wu R., Zhang Y., Zhu D. (2021). Acetoacetate promotes muscle cell proliferation via the miR-133b/SRF axis through the Mek-Erk-MEF2 pathway. Acta Biochim. Biophys. Sin..

[28] Yao Q., Li H., Fan L., Huang S., Wang J., Zheng N. (2021). The combination of lactoferrin and linolenic acid inhibits colorectal tumor growth through activating AMPK/JNK-related apoptosis pathway. PeerJ.

[29] Rosa L., Cutone A., Lepanto M.S., Paesano R., Valenti P. (2017). Lactoferrin: A Natural Glycoprotein Involved in Iron and Inflammatory Homeostasis. Int. J. Mol. Sci..

[30] Gao R., Watson M., Callon K.E., Tuari D., Dray M., Naot D., Amirapu S., Munro J.T., Cornish J., Musson D.S. (2016). Local application of lactoferrin promotes bone regeneration in a rat critical-sized calvarial defect model as demonstrated by micro-CT and histological analysis. J. Tissue Eng. Regen. Med..

[31] Reznikov E.A., Comstock S.S., Yi C., Contractor N., Donovan S.M. (2014). Dietary Bovine Lactoferrin Increases Intestinal Cell Proliferation in Neonatal Piglets. J. Nutr..

[32] Icriverzi M., Bonciu A., Rusen L., Sima L.E., Brajnicov S., Cimpean A., Evans R.W., Dinca V., Roseanu A. (2019). Human Mesenchymal Stem Cell Response to Lactoferrin-based Composite Coatings. Materials.

[33] Wei L., Wei L., Zhang X., Zhang X., Wang J., Wang J., Ye Q., Ye Q., Zheng X., Zheng X. (2019). Lactoferrin deficiency induces a pro-metastatic tumor microenvironment through recruiting myeloid-derived suppressor cells in mice. Oncogene.

[34] Wei L., Liu C., Wang J., Zheng X., Peng Q., Ye Q., Qin Z., Li Z., Zhang X., Wu Y. (2021). Lactoferrin is required for early B cell development in C57BL/6 mice. J. Hematol. Oncol..

[35] Zembron-Lacny A., Morawin B., Wawrzyniak-Gramacka E., Gramacki J., Jarmuzek P., Kotlega D., Ziemann E. (2020). Multiple Cryotherapy Attenuates Oxi-Inflammatory Response Following Skeletal Muscle Injury. Int. J. Environ. Res. Public Health.

[36] Yin H., Price F., Rudnicki M.A. (2013). Satellite Cells and the Muscle Stem Cell Niche. Physiol. Rev..

[37] Milner D.J., Cameron J.A. (2012). Muscle Repair and Regeneration: Stem Cells, Scaffolds, and the Contributions of Skeletal Muscle to Amphibian Limb Regeneration. Tuberculosis.

[38] Kargl C.K., Nie Y., Evans S., Stout J., Shannahan J.H., Kuang S., Gavin T.P. (2019). Factors secreted from high glucose treated endothelial cells impair expansion and differentiation of human skeletal muscle satellite cells. J. Physiol..

[39] Collins C.A., Olsen I., Zammit P.S., Heslop L., Petrie A., Partridge T.A., Morgan J.E. (2005). Stem Cell Function, Self-Renewal, and Behavioral Heterogeneity of Cells from the Adult Muscle Satellite Cell Niche. Cell.

[40] Saini J., McPhee J.S., Al-Dabbagh S., Stewart C.E., Al-Shanti N. (2016). Regenerative function of immune system: Modulation of muscle stem cells. Ageing Res. Rev..

[41] Zhang J., Han X., Shan Y., Zhang L., Du M., Liu M., Yi H., Ma Y. (2018). Effect of bovine lactoferrin and human lactoferrin on the proliferative activity of the osteoblast cell line MC3T3-E1 in vitro. J. Dairy Sci..

[42] Wang Z. (2021). Regulation of Cell Cycle Progression by Growth Factor-Induced Cell Signaling. Cells.

[43] Colao I., Pennisi R., Venuti A., Nygårdas M., Heikkilä O., Hukkanen V., Sciortino M.T. (2017). The ERK-1 function is required for HSV-1-mediated G1/S progression in HEP-2 cells and contributes to virus growth. Sci. Rep..

[44] Wang S., Wang X., Gao Y., Peng Y., Dong N., Xie Q., Zhang X., Wu Y., Li M., Li J. (2019). RN181 is a tumour suppressor in gastric cancer by regulation of the ERK/MAPK–cyclin D1/CDK4 pathway. J. Pathol..

[45] Meloche S., Pouysségur J. (2007). The ERK1/2 mitogen-activated protein kinase pathway as a master regulator of the G1- to S-phase transition. Oncogene.

[46] Ma Y., Fu S., Lu L., Wang X. (2017). Role of androgen receptor on cyclic mechanical stretch-regulated proliferation of C2C12 myoblasts and its upstream signals: IGF-1-mediated PI3K/Akt and MAPKs pathways. Mol. Cell. Endocrinol..

[47] Guo H.Y., Jiang L., Ibrahim S.A., Zhang L., Zhang H., Zhang M., Ren F.Z. (2009). Orally Administered Lactoferrin Preserves Bone Mass and Microarchitecture in Ovariectomized Rats. J. Nutr..

[48] Inubushi T., Kosai A., Yanagisawa S., Chanbora C., Miyauchi M., Yamasaki S., Sugiyama E., Ishikado A., Makino T., Takata T. (2020). Bovine lactoferrin enhances osteogenesis through Smad2/3 and p38 MAPK activation. J. Oral Biosci..

[49] Liu L., Jiang R., Liu J., Lönnerdal B. (2019). The bovine Lactoferrin-Osteopontin complex increases proliferation of human intestinal epithelial cells by activating the PI3K/Akt signaling pathway. Food Chem..

[50] Zhang Y., Wang X., Qiu Y., Cornish J., Carr A.J., Xia Z. (2014). Effect of indomethacin and lactoferrin on human tenocyte proliferation and collagen formation in vitro. Biochem. Biophys. Res. Commun..

[51] Jung H.-W., Choi J.-H., Jo T., Shin H., Suh J.M. (2019). Systemic and Local Phenotypes of Barium Chloride Induced Skeletal Muscle Injury in Mice. Ann. Geriatr. Med. Res..

