# Supplementary material for: Lactoferrin Deficiency Impairs Proliferation of Satellite Cells via Downregulating the ERK1/2 Signaling Pathway
Source: Int J Mol Sci. 2022 Jul 5;23(13):7478. doi: 10.3390/ijms23137478 (PMC9267821; doi:10.3390/ijms23137478)
Supplement: Supplementary file 1 [file ijms-23-07478-s001.zip › Supplementary Figure Legends.pdf]

Supplementary Figures

Titles and Legends of supplementary Figures

Supplementary Figure 1. Histopathological analysis of TA muscle after 7 and 60 days of $BaCl_2$ injury repair

A Representative photomicrograph of H&E-stained sections showing a delayed regeneration of injured TA muscle in *Ltf*-KO mice compared with that in WT at day 7 after $BaCl_2$ injection. Scale bar: 60 μm. **B** Statistical analysis of mean area of TA muscle cross-sections in adult *Ltf*-KO and WT mice 7 days after injury. **C** H&E staining analysis of transverse sections of the TA muscles from WT and *Ltf*-KO mice after recovery for 60 days. Scale bar: 60 μm. **D** Statistical analysis of mean area of TA muscle cross-sections in adult *Ltf*-KO and WT mice 60 days after injury.

Supplementary Figure 2. Schematic diagram of the targeting strategy of systemic knockout of *Ltf* in mice. **A** *Ltf* gene targeting strategy. The sgRNA was designed in intron 2 and intron 8, and exons 3-8 were deleted, resulting in a deletion of about 4 kb of the genome, thus achieving the knockout of the lactoferrin gene. **B** Genotyping of mouse tail genome agarose gel map. Mouse tails were protease digested for genome extraction and genotyped by PCR and agarose gel electrophoresis. The left picture is the agarose gel image of the amplified product by the WT primer, and the right image is the agarose gel image of the amplified product of the *Ltf*-KO primer. Lanes from left

to right represent water (negative control), heterozygote, WT, heterozygote, heterozygote, WT, *Ltf*-KO.

Supplementary Figure 3. Satellite cells grown in vitro for 4 days, and the detection of proliferation-related protein levels

A Left, the immunoblots presented here illustrate the protein levels of *p-AKT*, *AKT*, and *β-Tubulin* in SCs from WT and *Ltf*-KO mice grown for 4 days in vitro; right, quantification analysis the ratio of *p-AKT* / *AKT* from western blot bands, *β-Tubulin* as internal reference. **B** Left, the immunoblots presented here illustrate the protein levels of *p-JNK*, *JNK*, and *GAPDH* in SCs from WT and *Ltf*-KO mice grown for 4 days in vitro; right, quantification analysis the ratio of *p-JNK* / *JNK* from western blot bands, *GAPDH* as internal reference. **C** Left, the immunoblots presented here illustrate the protein levels of *p-p38*, *p38*, and *β-Tubulin* in SCs from WT and *Ltf*-KO mice grown for 4 days in vitro; right, quantification analysis the ratio of *p- p38* / *p38* from western blot bands, *β-Tubulin* as internal reference.
