# Supplementary material for: Lactoferrin Deficiency Impairs Proliferation of Satellite Cells via Downregulating the ERK1/2 Signaling Pathway
Source: Int J Mol Sci. 2022 Jul 5;23(13):7478. doi: 10.3390/ijms23137478 (PMC9267821; doi:10.3390/ijms23137478)
Supplement: Supplementary file 1 [file ijms-23-07478-s001.zip › Supplementary Table S2.pdf]

Table S2 Primer sequence of PCR

Primer	Sequence (5'-3')	Product size (bp)
WT-F	TGCTGGGATTCGAACTTGGCTGAAG	404
WT-R	AGACCGAGGAATCTGTGTGAGACCT	
Ltf-KO-F	CGCCGCTCAGTTGTGTCAAGAAATC	564
Ltf-KO-R	AGACCGAGGAATCTGTGTGAGACCT	
