# Supplementary figures and images for: Lactoferrin Deficiency Impairs Proliferation of Satellite Cells via Downregulating the ERK1/2 Signaling Pathway
Source: Int J Mol Sci. 2022 Jul 5;23(13):7478. doi: 10.3390/ijms23137478 (PMC9267821; doi:10.3390/ijms23137478)

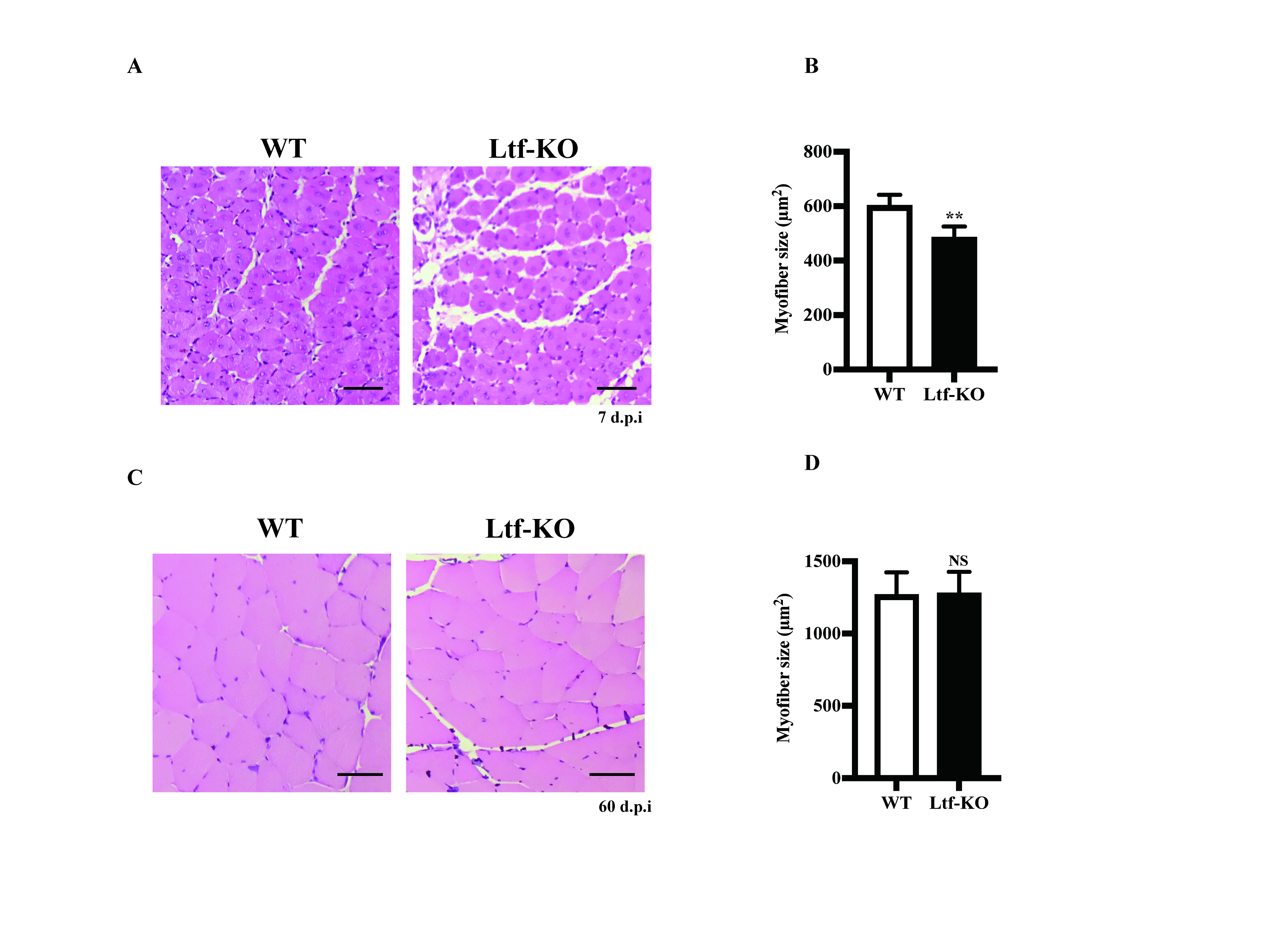

Supplement: Supplementary file 1 [file ijms-23-07478-s001.zip › Supplementary Figure S1.tif]

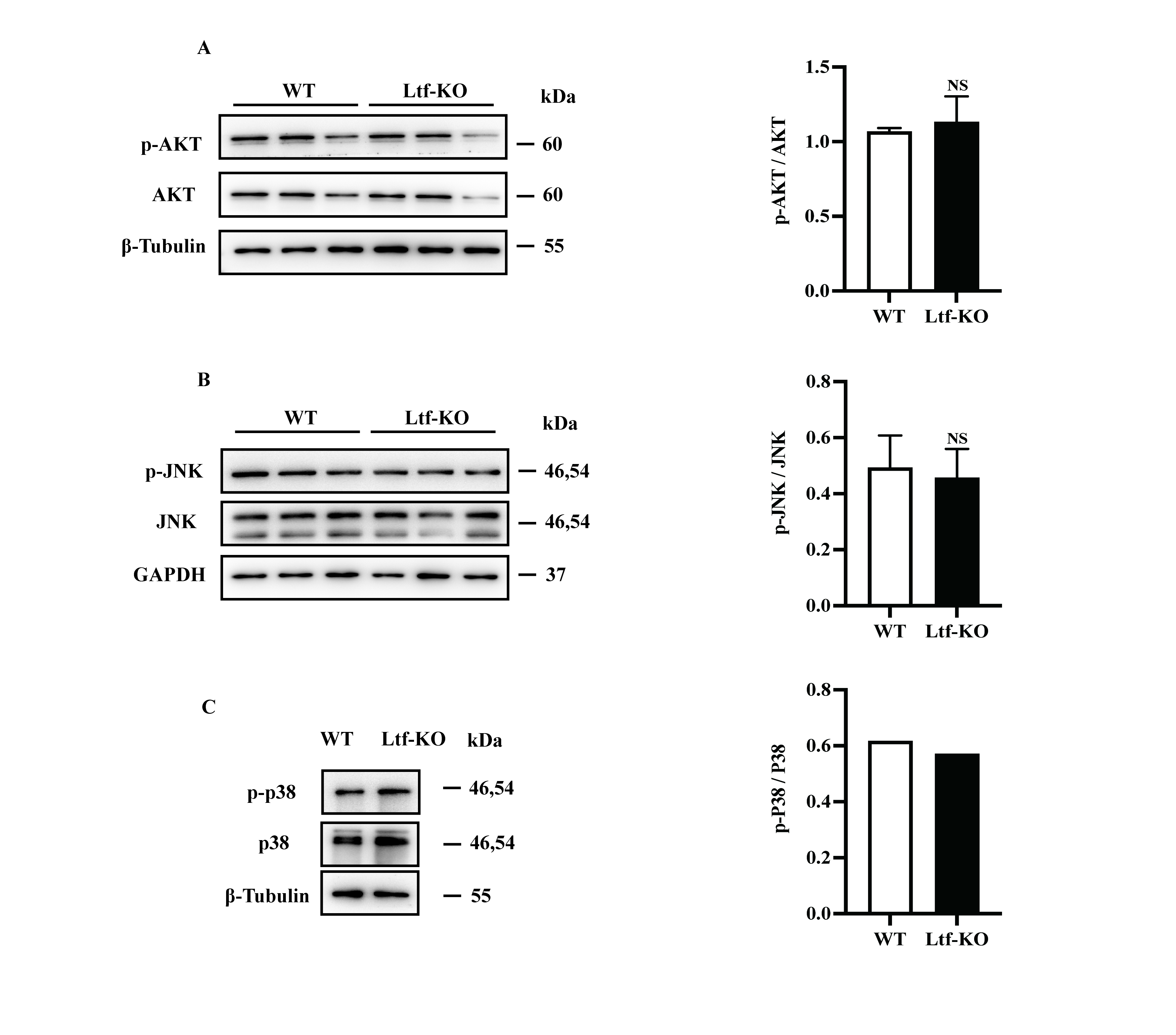

Supplement: Supplementary file 1 [file ijms-23-07478-s001.zip › Supplementary Figure S2.tif]
